# Daily Fluid Intake Behaviors and Associated Health Effects Among Australian and United States Populations

**DOI:** 10.3389/fspor.2022.898720

**Published:** 2022-06-09

**Authors:** Jesse N. L. Sims, Justin J. Holland, Travis Anderson, William M. Adams

**Affiliations:** ^1^Hydration, Environment, and Thermal Stress Lab, Department of Kinesiology, University of North Carolina at Greensboro, Greensboro, NC, United States; ^2^School of Exercise and Nutrition Sciences, Faculty of Health, Queensland University of Technology, Brisbane, QLD, Australia; ^3^Division of Sports Medicine, United States Olympic & Paralympic Committee, Colorado Springs, CO, United States; ^4^United States Coalition for the Prevention of Illness and Injury in Sport, Colorado Springs, CO, United States

**Keywords:** water, fluid intake, hydration, health, fluid intake behavior

## Abstract

Minimal data exist exploring intercontinental differences in fluid intake (FI) beliefs and behaviors and the impact on fluid intake practices (i.e., fluid intake volume, beverage type, and timing of fluid intake). Therefore, this study explored the impact that FI beliefs and behaviors had on FI practices among emerging adults living in the United States (USA) and Australia (AUS). A total of 489 individuals (74.5% female; USA, 79.4%; age, 25 ± 6 years completed a 23-item survey between November 2020 and June 2021). Participants detailed their FI practices. FI beliefs were evaluated to determine their contribution to FI behaviors across the day. Multinomial and multiple linear regression analyses explored the association of daily FI beliefs and behaviors across multiple domains. Independent sample *t*-tests and chi-square analyses were conducted to compare FI practices, beliefs, and behaviors between individuals in the USA and AUS. FI behaviors were significantly different between countries, with the USA more likely to consume fluids to meet a total target volume (β = 1.150, *p* = 0.036) and consume fluid at the same time as structured daily activities (β = 0.773, *p* = 0.046) compared to FI alongside food intake. However, there were no differences in the types of beverage consumed (juice, sugar-sweetened beverages, tea, and coffee), total fluid volume, and physical activity (PA) between countries (*p* > 0.05). Beverage consumption was higher among USA than AUS residents for water, beer, and wine (*p* < 0.05). Total fluid consumption was greater among males (3,189 ± 2,407 ml) than females (2,215 ± 1,132 ml; β = 3.61, *p* < 0.001), individuals who regularly consumed fluid during the day to meet a targeted volume (β = 1,728.5, *p* < 0.001), and those who regularly consumed fluid as a habitual behavior (β = 3.97, *p* < 0.001) compared to those individuals who only consumed fluid alongside mealtimes (β = 1,041.7, *p* < 0.001). FI behaviors differed between the USA and AUS; however, total volume consumed, type of beverage consumed, and FI beliefs were similar. FI practices and behaviors appear to be individualized and context-specific among the studied populations.

## Introduction

Water is a vital nutrient of the human body and is critical to the survival of human life through tightly controlled homeostatic mechanisms that affect physiological function. Fluid intake (FI) occurs *via* the consumption of water and various other fluids that contain other compounds, such as sugars and other sweeteners (sugar-sweetened beverages), caffeine, electrolytes, and alcohol. Compelling evidence suggests that meeting daily FI recommendations is associated with positive outcomes related to renal, cardiovascular, and metabolic health (de La Guéronnière et al., 2011; Enhörning et al., 2013, 2015; Sontrop et al., 2013; Carroll et al., 2015; Hooton et al., 2018). The perceptions of health risk have been demonstrated to have the strongest relationship with intentions to reduce consumption of sugar-sweetened beverages (SSB) (Dono et al., 2021), as intake of SSB is associated with increased risk for development of type 2 diabetes mellitus (Malik et al., 2010; de Koning et al., 2011; Malik and Hu, 2012), cardiovascular disease (Duffey et al., 2010; de Koning et al., 2012), obesity (Olsen and Heitmann, 2009; Chaloupka et al., 2011), and poor diet quality (Daniels and Popkin, 2010; Sharkey et al., 2011). Studies have suggested that limiting health risk behaviors can have a positive impact on health outcomes by preventing 80% of heart disease, cerebrovascular incidents, type 2 diabetes, and 40% of cancer (Baker, 2001; Ezzati et al., 2004).

Due to the differences in individual physiological requirements, values referencing adequate intake (AI) are used as the dynamic and complex nature of body water regulation, and homeostasis prohibits the determination of exact hydration levels. Fluid intake recommendations have been disseminated by many leading public health organizations (Grandjean, 2004; Institute of Medicine., 2005; EFSA Panel on Dietetic Products, Nutrition, and Allergies, 2010), but their varying recommendations reflect the innate nature of fluid requirements and individualization across varied environments. Global recommendations for FI were developed by the Institute of Medicine from a USA survey (2005) and recommend total fluid (drinking water, beverages, and fluid from foods) intake of 3.7 L/day for males and 2.7 L/day for females, respectively (Institute of Medicine., 2005; EFSA Panel on Dietetic Products, Nutrition, and Allergies, 2010). Approximately 20% of total fluid intake will come from fluids within foods, leaving the AI of drinking fluids to be amounts of 2.5 L/day for males and 2.0 L/day for females; this agrees with the recommendations set forth by the European Food Safety Authority (EFSA Panel on Dietetic Products, Nutrition, and Allergies, 2010). Findings from large cross-sectional studies surveying FI patterns and behaviors across targeted countries highlight water intake disparities across the lifespan (Gandy et al., 2018; Stookey and König, 2018; Iglesia-Altaba et al., 2021). Specifically, Stookey and König (2018) found among country differences in FI volume (range 1.7–2.3 L/day), composition, and FI patterns in those surveyed from Argentina, Brazil, Mexico, and Uruguay which should provide similar environmental conditions based on geography. These results highlight that current FI guidelines are not being followed, and further research is required to understand these barriers and why differences in fluid volumes occur in varied environments.

Data from the United States of America (US) indicate that sugar-sweetened beverage consumption is decreasing while water intake is increasing (Vieux et al., 2020), despite decreases in overall total FI in younger adults (Colburn and Kavouras, 2021); this may indicate a shift in FI behaviors related to fluid type or composition rather than volume consumption. This is in comparison with data from Australia (AUS) that highlighted a matched proportion of fluid volume (37%) from plain water and from other beverages, with the remainder being consumed *via* moisture from foods (Sui et al., 2016). This discrepancy may suggest that FI may be reflecting a focus on health-conscious behaviors as individuals begin to consume more plain water throughout their day. It also may suggest that the USA may be in transition toward the current behaviors implemented by Australians. Further investigations should consider whether the lag in time between behavioral adoption in the USA and AUS can be investigated by determining the underlying reasons why people consume fluids in varied types and volumes.

FI is highly individualized with many variables (e.g., body composition, physical fitness, goals, and social and cognitive health) driving FI behaviors in various environments. Textural elements of fluid (e.g., taste, viscosity, palatability, and temperature) may contribute to the consumption of a particular beverage type and guide preferences for FI behaviors (Baker and Jeukendrup, 2014). These behaviors may be influenced by prior knowledge, education, and physiological mechanisms that contribute to the cue toward fluid consumption. Fluid behaviors have been shown to be products of belief systems, suggesting that interventions targeting beliefs of FI should be targeted in the prevention of health outcomes (Winger et al., 2011). Therefore, understanding the beliefs behind fluid intake would enhance the understanding of the current mismatch between recommended guidelines and FI across the globe.

Current evidence shows the differences in habitual FI, both volume and beverage type, in adults and youth or adolescents across various countries around the world (Guelinckx et al., 2015a,b). The mechanisms surrounding FI behaviors is largely unknown, particularly as they pertain to health-related behaviors. Expansion of the knowledge of current FI behaviors will enable more targeted interventions across the spectrum of health and human performance and to ensure that the recommendations meet the current needs of all individuals. Similarly, improving our knowledge on FI behaviors will assist in improving the methodology of future FI research to account for both current and historical residency as these factors may influence current behaviors and response to intervention or change. Therefore, the purpose of this study was to characterize how FI beliefs and behaviors may influence FI practices (i.e., beverage consumption, fluid volume, and timing of fluid consumption) between individuals residing in the USA and AUS. The secondary aim of the study was to establish whether fluid intake recommendations are being met within this emerging adult population.

## Methods

Using a cross-sectional design, emerging adults (18–29 years) were recruited to complete an online survey (Qualtrics, Provo, UT, USA) to capture their FI beliefs and behaviors toward FI practices. University email listservs (University in the Southeastern USA), social media posts, and convenience sampling were used to recruit participants from November 2020 to June 2021. The study was intended to capture the practices employed by a diverse group of individuals with varying levels of daily energy expenditure, environmental conditions, health status, and occupations. Consent of the participants to complete this survey was implied by them clicking on the “I give consent to partake in the research study” button located at the bottom of the study information page before being able to view and complete the survey. All participants gave informed consent before undertaking the survey. This study was approved by the institutional review board at the University of North Carolina at Greensboro (#21-0132) and Queensland University of Technology (2000000945).

### Survey

To the authors' knowledge, there was no previous validated survey instrument to explore the research question. The lead investigator (JS) created an original questionnaire constructed from a variety of recent publications (Winger et al., 2011; Tyrwhitt-Drake et al., 2014; Ferreira-Pêgo et al., 2015), gaps in literature, and review articles in the domains of FI consumption, health-related behaviors, and FI perceptions.

The survey had three key components—(1) participant characteristics and demographics, (2) FI practices, and (3) FI beliefs and behaviors.

#### Participant Characteristics

The first section of the survey sought to capture a greater understanding of the studied population. Participants were asked to select their year of birth (age) and their sex. Participants were also asked to detail their current country of residence and postcode/ZIP code of that residence and the country where they spent >50% of their early years of life (up to the age of 18 years). These items were added to explore heritage and environmental changes related to geography. Physical activity was captured by participants detailing their frequency (total number of sessions per week) and duration (total minutes per week) for light, moderate, and vigorous activities, and participants were provided exemplar activities which was adapted from the World Health Organisation (2019). Physical activity was reported as a combined total of moderate and vigorous physical activity (MVPA). The last items in this section asked participants to indicate if they had a chronic health condition, and a descriptor of their occupation related to activity and environmental settings (e.g., active-indoors, in-active-outdoors).

#### Fluid Practices

The second section of the survey was designed to explore the current practices implemented in everyday living. Participants were provided with images, and information detailed the volume (in ml and ounces) of one serving of the following beverages: water, juice, sugar-sweetened beverages (e.g., soft drink and energy drinks), coffee, tea, wine, and beer. Participants were asked to indicate the amount of serves of each beverage consumed on average each day. Data were extrapolated to determine total fluid consumed for each beverage and total fluid intake over the day. Participants were asked to confirm consumption of beverage types (with extended examples) and included an option to indicate consumption of beverages not consumed.

Fluid frequency was assessed by participant indicating the most appropriate response: “I consume fluid predominately at meal times only,” “I consume fluid regularly during the day when I feel I need it,” “I consume fluid regularly throughout the day as I have a total target consumption that I aim to meet,” “I predominately only consume fluid when I have structured activities throughout my day (e.g., work meetings, gym sessions, mealtimes etc.),” “I consume fluid regularly throughout my day as it is a habit,” and “I don't know when I typically fluids.”

The role of fluid imbalance and risk of acute and chronic health was assessed by asking the participants to indicate which physiological systems (i.e., urological, gastrointestinal, heart and vascular health, neurological, respiratory disorders, hypertension, gallstones, and cancer) would have increased risk if fluid imbalances were to occur.

#### Fluid Intake Behaviors and Beliefs

FI behaviors and how they relate to physiological mechanisms were assessed using 5-point Likert scale (strongly-agree to strongly-disagree). The five items included: (1) individuals with chronic health conditions typically require alterations to their previous practices of fluid intake volumes and frequency, (2) I regularly wake up during the night and require the bathroom for urination, (3) I notice a difference in taste between types of water (e.g., bottle water, tap water, and filtered water), (4) Do you believe that lack of fluid intake impairs your cognitive function (e.g., alertness, memory, reaction time, perception, thinking, and decision-making)?, (5) My regular fluid intake regime has decreased as I have aged.

The final seven questions were structured as per the Precaution Adoption Process Model, and participants indicated their current beliefs toward the behavior from seven possible responses. Participants were asked their (1) belief about fluid volume consumption in relation to their physiological needs (body composition, physical activity, energy expenditure, digestion, elimination of waste products, and body temperature regulation) as well as a subsequent question on (2) how the types of fluid may also impact those physiological systems, (3) the interaction between fluid imbalances and acute health conditions, such as cognitive alertness, tiredness, alterations in mood, increased body temperature, and headaches, (4) their beliefs toward fluid imbalances leading to chronic health conditions, such as chronic kidney disease, high blood pressure, and respiratory disorders, (5) how the types of fluid may also contribute to chronic health conditions, (6) their beliefs about how fluid intake can impair sleep (7), and finally, how fluid intake behaviors may change with aging. All items related to fluid intake and physiology and fluid intake behaviors were displayed in a randomized order to minimize the effect of health model ordering influencing responses.

### Survey Validation

The survey went through a multistage content validation process by the authors and academics (*n* = 3) with expertise in the fields of hydration and human performance. The instrument was trialed among a group of 10 emerging adults with various education levels to ensure adequate readability. The final instrument comprised of 23 items using Likert scale and multiple-choice response options. Due to the novel nature of the instrument, a true power analysis was unable to be determined. However, similar hydration survey instruments have shown adequate response rates when the total sample size is >300 responses and/or five times the number of instrument items (i.e., 16 items = 80 responses) (Hosokawa et al., 2019). The research team aimed for a minimum of 100 responses per location to establish appropriate sample size. The final survey resulted in 23 items was developed to explore the FI practices, beliefs, and behaviors. However, the characterization of FI practices and achievement of current fluid intake recommendations is achieved using the first seven items of the survey.

### Data Reduction and Analysis

Analyses were performed in R (Version 3.5.5; R Core Team 2018) using the RStudio Environment (Version 1.0.143) and SPSS (IBM Corp. Released 2017. IBM SPSS Statistics for Macintosh, Version 26.0. Armonk, NY: IBM Corp). All data were presented as mean and standard deviation (SD) unless otherwise specified. The *t*-test and chi-square (χ^2^) analyses were used to assess the differences in fluid intake consumption (timing, volume, and type), physical activity, and outcomes of meeting FI recommendations between countries and sex differences within countries. Multinominal and multiple linear regression analyses were used to assess associations of fluid intake and associated beliefs and behaviors. All regression coefficients were presented relative to changes in fluid volume with their associated confidence intervals. Alpha was set at *p* < 0.05 for all analyses.

## Results

**Figure 1:**
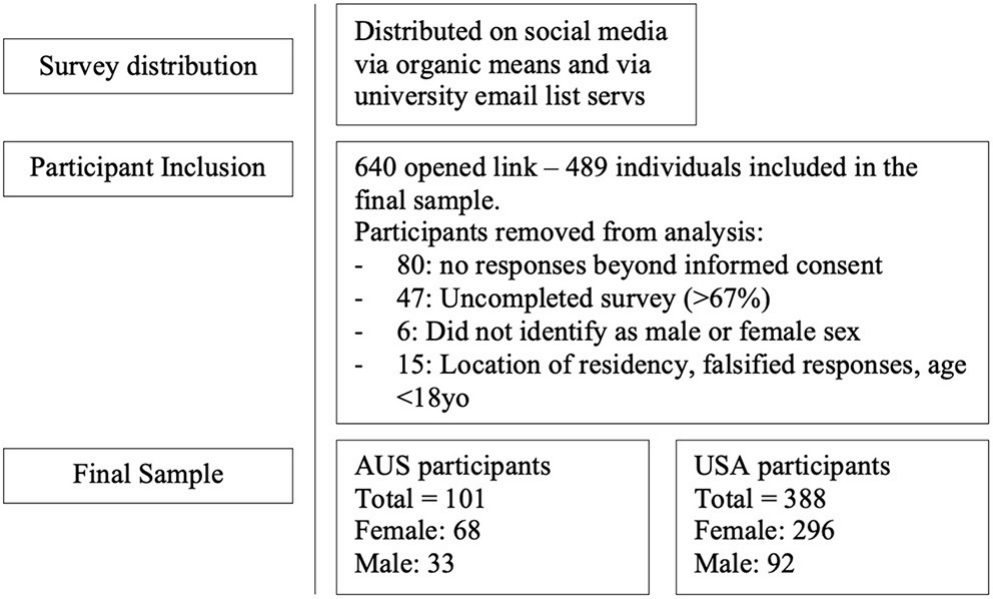
Participant inclusion in final sample based on response.

The survey was completed by 548 individuals with a total of 489 individuals included in the final sample. A total of forty-seven (8.6%) individuals were removed from the analysis due to non-completion of survey >67%. The cutoff score (>67%) was chosen as the final seven items of the survey were developed to reflect a health behavior model, and to ensure that adequate response to most survey items was completed. Individuals who did not identify as male or female sex were removed (*n* = 6, 1.1%) from the analysis due to the lack of adequate sample size. The remaining 15 (2.7%) participants were removed due to the reasons associated with location of residency, falsified responses (e.g., answering each question with same response, impossible values), or age <18 years (Figure 1). Participants (*n* = 3) who reported fluid intake volumes >7 L/day were only excluded from the analysis of fluid consumption evaluations as their reported values are two times greater than recommended values and likely an error in self-reporting. Participant demographics are detailed in Table 1 and their reported fluid consumption in Table 2.

**Table 1:** Participant demographics (*n* = 489)^a^.

	**Group**		
**Item**	***AUS (n = 101)***	***USA (n = 388)***	
Age (years)	24 ± 3.9	27 ± 6.8	
Sex	• Female: *n* = 68 (67.3%) Male: *n* = 33 (32.7%)	• Female: *n* = 296 (76.3%) • Male: *n* = 92 (23.7%)	
MVPA^b^ (mins.week^−1^)	415 ± 1,055	250 ± 435	
Self-reported chronic health condition	• Yes: 13 (12.9%) • No: 87 (86.1%)	• Yes: 67 (17.3%) • No: 320 (82.5%)	
Reported occupation activity level description	• In-active indoors: 36 (35.6%) • In-active outdoors: 1 (1%) • Active indoors: 50 (49.5%) • Active outdoors: 14 (13.9%)	• In-active indoors: 179 (46.1%) • In-active outdoors: 5 (1.3%) • Active indoors: 150 (38.7%) • Active outdoors: 54 (13.9%)	
Meeting current fluid intake guidelines	• Female: *n* = 33 (48.5%) Male: *n* = 18 (54.5%)	• Female: *n* = 143 (48.3%) • Male: *n* = 54 (58.7%)	

*^a^Data presented as mean (standard deviation)*.

*^b^Moderate–vigorous physical activities*.

**Table 2:** Daily reported fluid intake (ml) as mean (SD) of the sample population.

	**AUS**			**USA**			**Total**		
	**Female (*n* = 68)**	**Male (*n* = 33)**	**Total (*n* = 101)**	**Female (*n* = 296)**	**Male (*n* = 92)**	**Total (*n* = 389)**	**Female (*n* = 364)**	**Male (*n* = 125)**	**Total (*n* = 489)**
**Total fluid**									
Mean (SD)	2,168 ± (973)	2,827 ± (1,120)	2,383 ± (1,064)	2,225 ± (1,165)	3,319 ± (2,718)	2,485 ± (1,729)	2,215 ± (1,131)	3,189 ± (2,407)	2,372 ± (1,223)
**Water**									
Mean (SD)	1,577 ± (816)	2,242 ± (1,110)	1,796 ± (970)	1,343 ± (848)	2,153 ± (2,100)	1,535 ± (1,305)	1,387 ± (846)	2,176 ± (1,886)	1,585 ± (1,248)
**Juice**									
Mean (SD)	51 ± (102)	83 ± (173)	62 ± (129)	75 ± (162)	126 ± (280)	87 ± (197)	70 ± (153)	115 ± (256)	81 ± (185)
**Coffee**									
Mean (SD)	258 ± (323)	398 ± (429)	304 ± (365)	351 ± (380)	448 ± (598)	375 ± (443)	334 ± (371)	435 ± (557)	360 ± (428)
**SSB**									
Mean (SD)	78 ± (159)	68 ± (122)	75 ± (148)	155 ± (334)	241 ± (414)	178 ± (356)	141 ± (310)	195 ± (368)	154 ± (326)
**Beer**									
Mean (SD)	0	8 ± (49)	3 ± (28)	24 ± (134)	88 ± (164)	40 ± (144)	20 ± (121)	67 ± (147)	31 ± (129)
**Wine**									
Mean (SD)	11 ± (54)	5 ± (26)	9 ± (47)	25 ± (73)	16 ± (75)	23 ± (73)	22 ± (70)	13 ± (66)	19 ± (69)
**Tea**									
Mean (SD)	215 ± (421)	23 ± (91)	152 ± (360)	251 ± (789)	246 ± (670)	250 ± (761)	244 ± (733)	188 ± (584)	230 ± (698)

### Fluid Intake Practices

**Figure 2:**
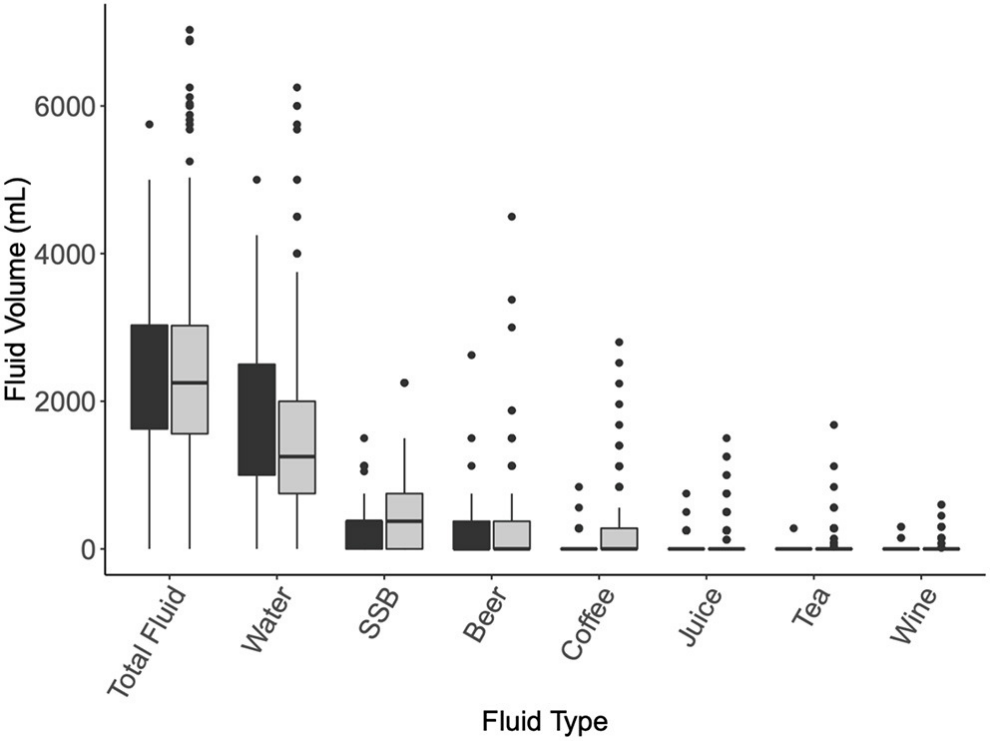
Fluid intake volume [Mean (SD)] by beverage type. Assessed by Independent sample *t*-tests and chi-square analyses. USA, black; SSB, sugar-sweetened beverages.

Fluid intake recommendations were met by 58% (*n* = 54) of males and 48% (*n* = 143) of females in the USA, respectively. Similarly, 54% (*n* = 18) of males and 48% (*n* = 33) of females in the AUS sample population met the AUS fluid intake guidelines (Table 1). FI recommendations were met across countries and sex, with a significant difference in FI among the USA participants based on sex [female = 2,225.23 ± 1,165 ml; *t*_(295)_, 3.324; *p* ≤ 0.001 and male 3,319.30 ± 2,717 ml; *t*_(91)_, 2.891; *p* = 0.002]. Furthermore, total FI in this USA sample population was statistically greater than the current USA FI guidelines [χ^2^ (167, *N* = 367) = 344.91, *p* ≤ 0.001]. Total FI was greater among all males (3,189.36 ± 2,406 ml) than females [2,214.53 ± 1,131 ml; *t*_(487)_, 6.033; *p* ≤ 0.001; Table 2]. Total FI was similar between the USA and AUS [*t*_(487)_, 0.562; *p* = 0.287; Figure 2].

There were no differences in the type of beverage consumed (juice, sugar-sweetened beverages, tea, and coffee), total fluid volume, PA, or FI beliefs between countries (*p* > 0.05, Figure 2). Beverage consumption was higher among USA than AUS residents for beer [MD = 36.68 (95% CI; −64.90, −8.46); *p* = 0.005] and wine [13.74 (−28.83, 1.35); *p* ≤ 0.001]. Plain water intake contributed to an average total of 1,585 ± 1,248 ml across the participants and was significantly different between locations with greater consumption in USA participants [*t*_(487)_, 1.751; *p* = 0.040].

Fluid volume [2,662 ± 1,444 ml; *t*_(487)_, 2.660; *p* = 0.008] and water intake [1,796 ± 1,156 ml; *t*_(487)_, 3.679; *p* < 0.001] were significantly higher with those who completed >150 min of MVPA per week.

### Fluid Intake Behaviors and Beliefs

**Figure 3:**
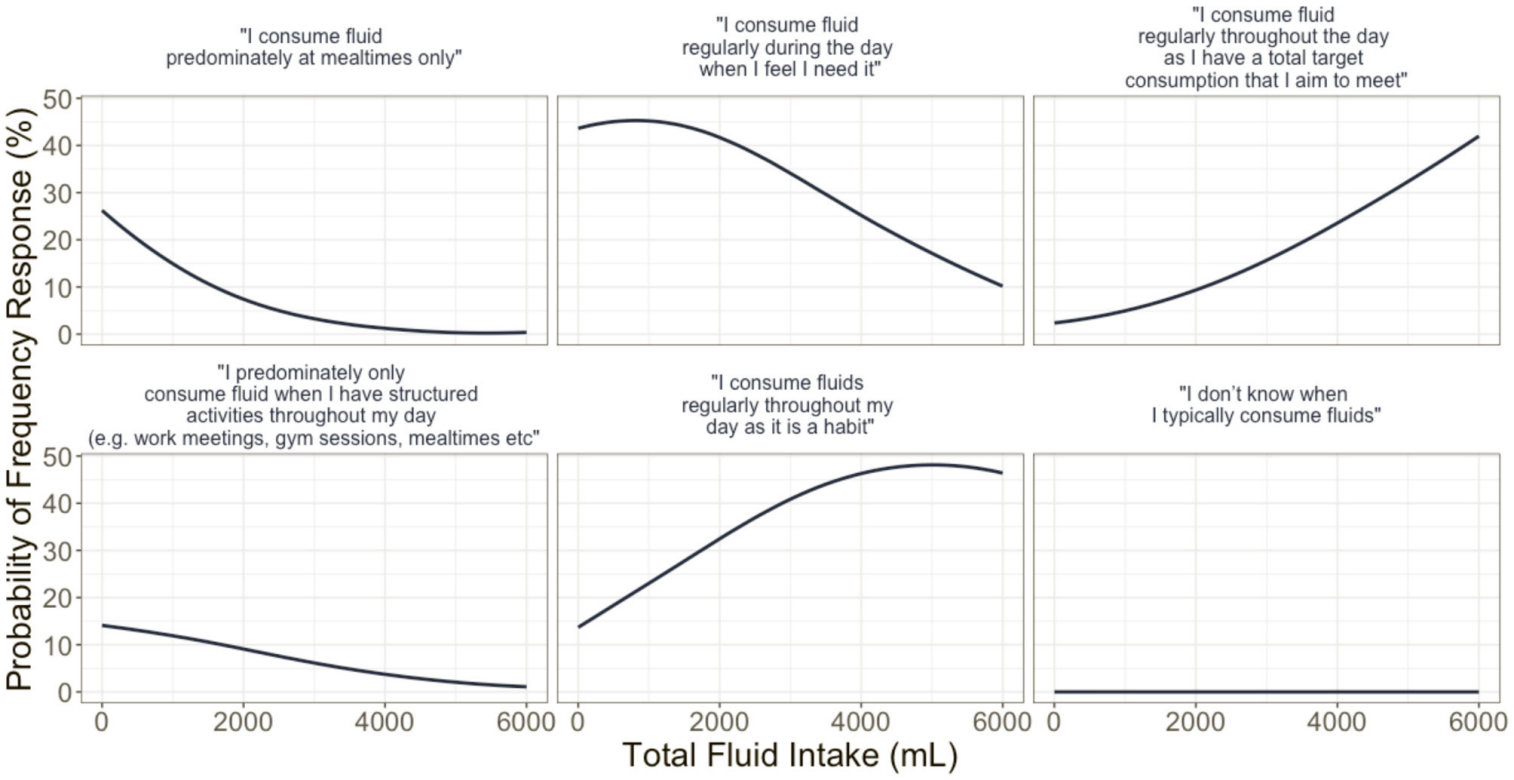
Multinomial model derived probabilities of fluid intake responses to the question on fluid frequency, given total fluid intake after controlling for country, gender, moderate–vigorous physical activities, and chronic health condition.

**Figure 4:**
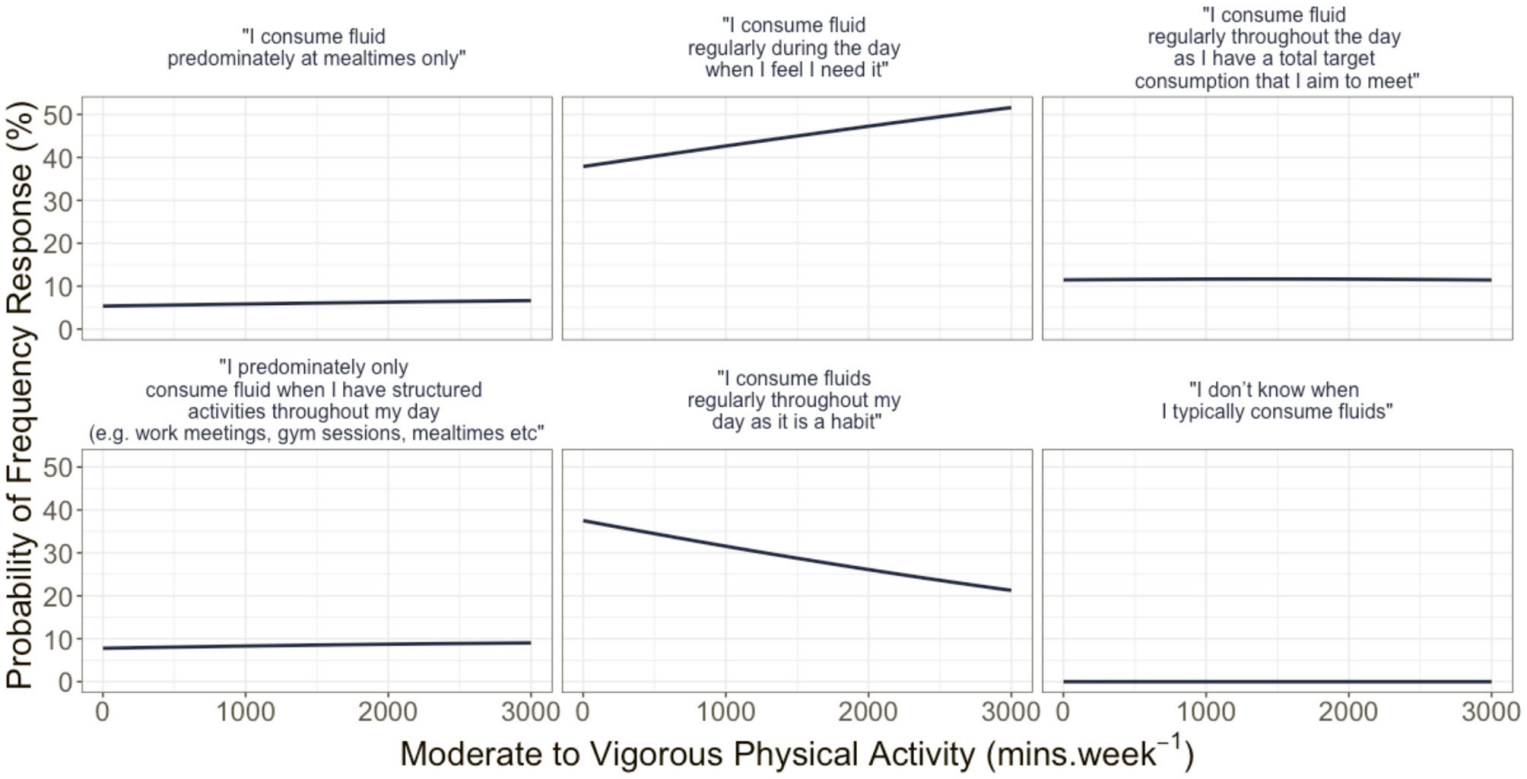
Multinomial model derived probabilities of fluid intake responses to the question on fluid frequency, given moderate–vigorous physical activities after controlling for country, gender, total fluid intake, and chronic health condition.

FI behaviors differed between countries with the USA more likely to consume fluids to meet a total target volume (β = 1.150, *p* = 0.036) and consume fluid at the same time as structured daily activities (β = 0.773, *p* = 0.046) compared to FI alongside food intake. The probability of fluid frequency response is delineated in Figure 3 and further derived in Figure 4 in reference to minutes of MVPA per week.

Participants were asked to describe their frequency of FI across an average day. USA participants (*n* = 146, 37.5%) reported that their FI frequency typically reflects when they believe they require fluid, compared to reaching a pre-planned volume target (*n* = 49, 12.6%). AUS participants indicated a similar trend with FI frequency (*n* = 41, 40.6%) and targeted FI volume (*n* = 9, 8.9%), respectively. Individuals with chronic health conditions were less likely to be aware of their frequency of FI (β = 1,880.9, *p*=0.001).

## Discussion

The aim of this investigation was to characterize how FI practices are shaped by FI beliefs and behaviors among USA and AUS residents. This study was novel in that it addressed FI practices by a specifically developed survey targeting current behaviors and beliefs toward FI, with the presented data reporting on how the FI practices may be related to FI behaviors. The results found that FI behaviors were not the same in both locations, despite the similarities of western culture. There were similarities in the type and volume of fluids consumed which included reported values of total intake that were in excess of the FI recommendations set by the respective national health organizations.

Response to FI frequency item showed that increased consumption of fluid best reflects those who consume fluids regularly throughout the day as a habit, and those who have a total target that they aim to meet. The probability of participants indicating their consumption of fluid in response to the perception that they needed it was greatest at total fluid intake volume of <2,000 ml. The probability of this response when controlling for MVPA displayed a positive linear response (Figure 3). Similarly, the probability of reporting behavior related to consumption due to habitual behavior had a negative correlation to FI. The findings related to fluid frequency, when controlling for country, sex, physical activity, and reported chronic health condition, highlight that FI behaviors are highly variable, with the timing of FI best reflecting overall health-related behaviors rather than reflective of set time points, activities, and engagements. However, the large variability in reported total daily FI and the average consumption in excess of the current recommended FI guidelines may require future interventions to explore FI across the whole day, and multiple days, and how these behaviors may change across time points of the day and in different environments.

Plain water intake was similar across locations with an average intake of 1,585±1,248 ml. These volumes of water intake reflect an average of 74 (AUS) and 72% (USA) consumption of water from the total percent FI reported. These values are much greater than the 37% contribution of water to total FI that was recently described in Australia (Sui et al., 2016). Similarly, these discrepancies display a difference in the behavioral practices undertaken by this sample population as water consumption is much greater than other consumption of other fluid properties. This may suggest that these individuals could be exhibiting more health-related practices by preferencing water over other fluid types, and that the focuses on health-related behavior choices are now being adopted in both AUS and USA. The analysis by Sui et al. (2016) was published ~5 years prior to when the present data were collected. The USA Department of Health and Human Services is required to release dietary guidelines for Americans every 5 years. However, there has not been a change in the AI levels of fluid intake despite obvious discrepancies in behavioral practices and consumption of water intake during this time. The extensive review process of the 2013 AUS guidelines is currently being undertaken (2022), which highlights an even greater time difference for the recommended guidelines. A more frequent review of FI recommendations should be undertaken. The FI recommendations would benefit from the inclusion of reviewing and understanding the behavioral trajectory of these practices and how they may influence current and future FI recommendations.

There were no significant differences between types of fluids consumed between countries or gender. This may be explained by the age of the participants and financial implications related to their current life status. The survey was primarily distributed to college or University (tertiary education) age students, whereby the minimum legal age for consumption of alcohol is 21 years in the USA (18 years for AUS), and students are typically bound to the meals and food services of their respective universities in the USA. AUS living arrangements are typically different whereby the population is bound by cost of living, food availability, and food supply. However, the survey was targeted at college participants to explore the emerging adult population, although confirmation of college attendance was not collected. Therefore, this highlights that the general patterns of behavior related to fluid choice are not only a reflection of accessibility to varied fluid types. If we are wanting to focus on the promotion of health benefits related to water consumption and decrease of sugar-sweetened beverages, then age brackets (such as this emerging adult population) may be appropriate. This is in the consideration of the reduction of many competing factors (i.e., accessibility, affordability, providing for others, social norms, etc.,) and therefore reduced number of barriers to overcome toward water selection as a preferred beverage choice.

It should be expected to see a greater increase in FI among those who are more physically active to reflect the physiological requirements and processes associated with these activities. This was observed in this study with fluid volume [2,662 ± 1,444 ml; *t*_(487)_, 2.660; *p* = 0.008] and water intake [1,796 ± 1,156 ml; *t*_(487)_, 3.679; *p* < 0.001] greater in those who completed >150 min of moderate–vigorous physical activities per week. Observational trends in those who meet the Physical Activity Guidelines for Americans show a 9.6% increase from 1998 to the latest report released by the CDC in 2018 (Hyde et al., 2021). Increases in total FI and shifts in fluid preference to water intake may be related to an increase in physical activity. However, despite a small increase in adequate physical activity participation, most adults do not meet physical activity guidelines. This may suggest that targeting FI in conjunction with physical activity recommendations may attenuate the improvements in overall health behaviors, but physical activity is not a likely driver of the observed changes in FI behaviors.

Government policy, media, marketing, corporations, education, and socioeconomic status all influence the behaviors on healthy FI. In this study, the results indicated that sugar-sweetened beverage intake was lower than previously reported with water intake increasing. In alignment with our results, there has been a trend in more recent times for reduced sports drink consumption in adolescents (Cordrey et al., 2018). Though in other countries, predominately low energy drink sales and associated diseases, including heart disease, obesity, and diabetes, have been on the rise (Stacey et al., 2017). Advertising in the form of television, radio, and print and more recently social media with the engagement of influencers and celebrities has had a strong influence on the way in which adolescents and emerging adults select and consume beverages (Kucharczuk et al., 2022). Parental behaviors and knowledge of appropriate fluid guidelines may also impact adolescent fluid behaviors with adolescent SSB intake associated with a higher intake of SSB in parents (Lundeen et al., 2018). Outside of media and social constructs, environmental changes to University dining halls that promote healthy beverage intake by coloring coding choices and advertising healthier options have been successful in increasing water uptake while lowering SSB (Di Sebastiano et al., 2021). Taxation on SSB's in some countries has substantially driven down the use of SSB though the improvements on body composition may be small (Gracner et al., 2022) with the percentage of taxation, country, socioeconomic status, age, and sex being constructs that affect the effectiveness of SSB taxation (Acton et al., 2021).

This study presents novel and important findings on FI behaviors across a large and demographically heterogeneous sample. However, some limitations should be acknowledged. First, the survey deployed in this study was designed and piloted using sound survey-development methodology and was reviewed for content validity by several experts; no criterion-related validity studies were employed prior to this study. Therefore, some questions within the present survey may be measuring similar yet not identical constructs to those intended and interpreted by the researchers herein. Given this study's interesting and actionable results, it is recommended that future researchers conduct further validity and reliability studies on the survey tool. Second, these survey data are inherently subjected to all general limitations of survey responses, including social desirability bias. This may have been limited *via* the online deployment of the survey, but nevertheless should be considered. Finally, no attempt was made to target any specific regions in the USA or AUS, although, due to the distribution channels used, the results may be biased toward more metropolitan areas and potentially biased toward geographical areas on the east coasts of both USA and AUS. This is particularly relevant for FI research, as geography and thus environmental considerations will impact FI behaviors. Moreover, metropolitan centers likely have differential access to fluid compared to more rural parts of the country. Therefore, generalization of these results across the entire country is cautioned, and future studies should consider sub-analyses by country region and population density.

## Conclusion

FI behaviors differed between the USA and AUS; however, total volume consumed and type of beverage were similar. Fluid intake practices and behaviors appear to be individualized and context-specific among the studied populations. Further research is required to understand how fluid selection and frequency behaviors are changing over time, and across locations, despite current reported FI still exceeding the current recommendations. Additional work is required to determine whether the current recommendations are adequate and whether the timing of investigation and disseminations of the guidelines reflects current practices. The presented work could suggest that more specific beverage recommendations (i.e., type and volume of beverage consumed) should be considered as this could assist in improving overall health outcomes which is the ultimate basis of FI recommendations.

## Data Availability Statement

The data is available upon reasonable request.

## Ethics Statement

The studies involving human participants were reviewed and approved by University of North Carolina at Greensboro and Queensland University of Technology. The patients/participants provided their written informed consent to participate in this study.

## Author Contributions

JS, JH, and WMA contributed to study design, data collection, and interpretated the data. JS and TA contributed to data analysis and data visualization. All authors contributed to critical revisions of the manuscript and have read and agreed to the published version of the manuscript.

## Author Disclaimer

This work was the authors' own and not that of the United States Olympic and Paralympic Committee, or any of its affiliates or members.

## Conflict of Interest

The authors declare that the research was conducted in the absence of any commercial or financial relationships that could be construed as a potential conflict of interest.

## Publisher's Note

All claims expressed in this article are solely those of the authors and do not necessarily represent those of their affiliated organizations, or those of the publisher, the editors and the reviewers. Any product that may be evaluated in this article, or claim that may be made by its manufacturer, is not guaranteed or endorsed by the publisher.
